# Improving Medical Student Inpatient Documentation Through Feedback Using a Note Assessment Tool

**DOI:** 10.7759/cureus.23369

**Published:** 2022-03-21

**Authors:** Michelle Kim, Neilson Chan, Jonathan Evans, Jonathan K Min, Amy C Hayton

**Affiliations:** 1 Internal Medicine, University of New Mexico, Albuquerque, USA; 2 Internal Medicine, Loma Linda University, Loma Linda, USA; 3 Psychology, Loma Linda University, Loma Linda, USA; 4 Internal Medicine, University of California Irvine Medical Center, Orange, USA; 5 Internal Medicine, Loma Linda VA (Veteran Affairs), Loma Linda, USA

**Keywords:** feedback, internal medicine clerkship, note assessment tool, student documentation, progress notes

## Abstract

Introduction

Documentation within the Electronic Health Record (EHR) is an essential skill for medical students to succeed in residency and post-residency training. The increased use of medical student progress notes for billable services raises the need for the education and assessment of quality note writing. We hypothesized that structured note feedback using a note assessment tool would improve the quality of medical student inpatient progress notes.

Methods

We conducted a retrospective study to review the quality of student inpatient progress notes written before and after structured feedback using the Responsible Electronic Documentation (RED) checklist throughout a third-year internal medicine clerkship. The first intervention group received feedback from clerkship directors in the 2017-2018 academic year and the second intervention group received feedback from ward residents/attendings in the 2018-2019 academic year. Within each intervention group, the total note scores from pre and post-intervention were compared.

Results

Feedback from clerkship directors yielded a greater increase in students’ total note score from pre to post-intervention compared to ward resident/attending feedback (F(1,255) = 12.84, p < 0.001). Cohen’s d effect size value was greater for the clerkship director feedback arm (d=0.71) compared to the ward resident/attending feedback arm (d=0.24). Post-hoc analyses using dependent sample t-tests revealed that there were significant increases in total note scores from pre to post-intervention for both the clerkship director arm (t(123) = 8.26, p < 0.001, d = 0.71) and the ward resident/attending arm (t(132) = 2.85, p = 0.005, d = 0.24).

Conclusion

Clerkship director feedback led to a greater increase in medical student documentation compared to ward attending/resident feedback. Nonetheless, structured feedback with a note assessment tool, whether from clerkship directors or ward attendings/residents, leads to a significant improvement in medical student documentation. Though there are various methods for providing feedback, educators can use the RED checklist to provide clear guidelines that will facilitate note-writing feedback.

## Introduction

Clinical documentation within Electronic Health Records (EHR) plays a key role in patient care and healthcare systems. It creates a record of patient encounters, serves as a device for communication between providers, streamlines coding and billing, and acts as a tool for further safety and quality improvement. Given its fundamental purpose, medical educators agree that documentation within the EHR is an essential skill for students to succeed in residency and post-residency training [1-3]. Although both the Association of American Medical Colleges (AAMC) and the Accreditation Council for Graduate Medical Education (ACGME) stress the importance of documentation skills within the EHR [2-3], the majority of note-writing instruction and student experience remains highly variable.

A 2012 survey of clerkship directors by the Alliance for Clinical Education found that only 64% of programs allowed students to access the EHR; of these programs, only two-thirds allowed students to write notes for entry into the EHR [4]. A nationwide survey conducted between 2012 to 2016 by the Liaison Committee on Medical Education (LCME) found similar results, with students entering notes in less than 65% of their third-year clerkships [5].

The limited use of medical student notes in the EHR is partially reflective of previous Centers of Medicare & Medicaid Services (CMS) guidelines restricting the elements of a billable note that could be authored by a medical student. With new revisions released by CMS in 2018, teaching physicians can now use medical student documentation for billable services with appropriate supervision [6]. With these changes, student documentation within the EHR is projected to increase. In doing so, two important questions are raised: (1) “Do medical students write quality notes?” and (2) “can medical students be taught to write quality notes?”

Though there is a paucity of literature regarding the objective quality of medical student notes, available studies show that student documentation of patient encounters is often inaccurate, copy-pasted, or filled with “note bloat” [7-10]. One of the main reasons for the poor quality of medical student notes in past studies may be due to the lack of sufficient and consistent feedback [11]. Effective feedback has been shown to be an important tool for increasing the quality of physician and student clinical performance [12-15]. However, most of these studies on student note quality involved standardized patients or simulated environments, which may not reflect real patient-doctor encounters in an inpatient or clinical setting [8,16-17].

We set out to investigate whether student documentation with non-standardized patients could improve with feedback and hypothesized that structured note feedback using a note assessment tool would improve the quality of medical student inpatient progress notes.

## Materials and methods

We conducted a retrospective study at Loma Linda University School of Medicine (LLUSOM) between the 2017-2018 academic year and the 2018-2019 academic year to review the quality of inpatient student progress notes. The entire class of 163 third-year medical students during the 2017-2018 academic year and the entire class of 155 third-year medical students during the 2018-2019 academic year participated.

At our university, students on their 10-week core Internal Medicine (IM) clerkship rotate at two of five different hospital sites, splitting the clerkship into two inpatient blocks. We conducted the intervention (note feedback) at Week 5 of the 10-week rotation. All students were required to turn in two progress notes for evaluation, one before the intervention and the other after the intervention. 

The Responsible Electronic Documentation (RED) checklist was chosen to evaluate the quality of notes. This tool was developed by the Northwestern University Feinberg School of Medicine as a way to evaluate inpatient progress notes (Appendix) [18]. The RED checklist takes minimal time to complete (on average 7-9 minutes), does not require prior knowledge of the patient, discourages copy and pasting from previous notes, and rewards critical reasoning on the assessment/plan portion of the note by the way the points are distributed in the checklist. Other tools, such as PDQI-9, QNOTE, HAPA, and Audit tool (from U of Wisconsin), were considered for use in our study [19-22]. However, these tools either did not focus on inpatient notes or had mixed results with a large group of students in improving note quality [22-24].

We compared the quality of student notes before and after two interventions: feedback by clerkship directors in the 2017-2018 academic year and feedback by ward residents/attendings in the 2018-2019 academic year.

Feedback by clerkship directors

For the first intervention in the 2017-2018 academic year, one of three IM clerkship directors evaluated notes with the RED checklist and then gave feedback during an individual scheduled 15-minute session at mid-rotation Week 5. Interrater reliability was not measured, but clerkship directors had a group training session on how to use the RED checklist to evaluate student notes and determined consensus on how they interpreted the RED checklist. IM clerkship directors had no previous knowledge about the patients being discussed in the note allowing their evaluation to be based solely on the checklist for an accurate, responsible note.

Feedback by ward residents/attendings

For the second intervention in the 2018-2019 academic year, residents and attendings responsible for the same patients as the students provided feedback. They also used the RED checklist to evaluate notes and gave feedback informally during a regular workday at mid-rotation Week 5. The ward residents/attendings did not have any previous training on how to use the RED checklist other than instructions that were written on the checklist itself.

At the end of the rotation (Week 10), both intervention groups had their notes graded using the RED checklist. Students in the first intervention had the second set of notes graded by IM clerkship directors, whereas students in the second intervention had the second set of notes graded by their new ward residents/attendings on their second block of the clerkship.

Data analytic plan 

Inclusion criteria included students who had a complete set of data: two inpatient progress notes and two graded RED checklists, from mid and end-of-rotation. In order to examine the efficacy of the interventions in improving students’ note writing, we conducted a mixed-design analysis of variance (ANOVA) to determine whether the changes in students’ scores on the RED checklist were different depending on the intervention group. A reverse-score square root transformation was performed on the RED checklist data to correct for non-normally distributed data and violation of the assumption of homogeneity of variances for ANOVA. Cohen’s d as an effect size was used to measure the magnitude of change in RED checklist scores before and after the intervention. Post-hoc dependent sample t-tests were conducted with each intervention group to determine whether there were significant changes in RED checklist scores before and after the intervention for each group. All analyses were conducted in IBM SPSS 20 (IBM Corp. Armonk, NY).

We received institutional IRB exemption for this study.

## Results

For intervention 1 (clerkship director feedback), 124 out of 163 students (76%) met inclusion criteria and were included in the analysis. For intervention 2 (ward resident/attending feedback), 133 out of 155 students (85.8%) met inclusion criteria and were included in the analysis.

Differences in RED checklist scores between pre and post-intervention by feedback from clerkship directors vs. ward resident/attendings

For intervention 1, the mean RED checklist score was 75% pre-intervention compared to 86% post-intervention. For intervention 2, the mean RED checklist score was 90% pre-intervention compared to 93% post-intervention.

Results from the mixed-design ANOVAs indicate that the increase in the RED checklist scores was significantly greater from pre to post-intervention for students who received feedback from clerkship directors compared to students who received feedback from ward resident/attendings on the total RED checklist score (F(1,255) = 12.84, p < 0.001). Post-hoc analyses using dependent sample t-tests revealed that there were significant increases in RED checklist scores from pre to post-intervention for both the clerkship director arm (t(123) = 8.26, p < 0.001, d = 0.71) and the ward resident/attending arm (t(132) = 2.85, p = 0.005, d = 0.24). Additionally, the results from the ANOVA indicate that when collapsing across time-points, those in the clerkship director arm were rated significantly lower on average (M=0.90) using the RED checklist than those in the ward resident/attending arm (M=0.75) (p < 0.001). Results and descriptive statistics are summarized in Table 1.

**Table 1:** Difference in RED checklist score from pre to post-intervention depending on clerkship feedback or ward resident/attending feedback ***p < .001. Note. Scores on the RED checklist were collected at pre and post-intervention. Analysis was performed on reverse-second square root transformed data to correct for non-normally distributed data. Arithmetic means and SDs are presented in this table for ease of interpretation. M = mean. SD = standard deviation. d = Cohen’s d effect size. TG = treatment group; T = time. The TG x Time interaction was based on df = 1, 255.

	Treatment Group						
	Intervention 1: Clerkship Director Feedback (n=124)			Intervention 2: Ward Resident/Attending Feedback (n=133)			
	Pre	Post		Pre	Post		
	M(SD)	M(SD)	d	M(SD)	M(SD)	d	F(TG x T)
Total	.75(.14)	.86(.12)	0.71	.90(.11)	.93(.08)	0.24	12.84***

## Discussion

Our results found that feedback from clerkship directors yielded a greater increase in students’ total note scores from pre to post-intervention and had a larger effect size (d=0.71) compared to ward resident/attending feedback (d=0.24). The results also showed that students’ pre-intervention total note scores were lower, on average, when graded by clerkship directors (M=0.75) compared to ward residents/attendings (M=0.90). We propose four potential reasons for the different findings between the two groups:

1. The Quality of Feedback Between the Two Groups Was Likely Non-Identical

Clerkship directors have much more experience giving feedback to students than residents and most attendings. In the second intervention, while some of the evaluators were attendings, most were residents (68-74% residents, 18-22% by in-house attendings, and 7-8% by unknown evaluators). Clerkship directors are likely more effective teachers with more experience giving feedback compared to residents who lack experience and instruction on giving effective feedback [25-26].

2. The Difference in Knowledge/Utilization of the​​​​​​​ RED Checklist by the Two Interventions

More experience with the assessment tool likely contributed to the larger increase of total note scores from pre to post-intervention and the larger effect size in the clerkship director arm. Though the RED checklist is able to be utilized by individuals using it for the first time, those who are unfamiliar with the RED checklist will need to consult a detailed key to grade progress notes [18]. Interrater reliability was not measured, but all clerkship directors met to establish their interpretation of how to grade and create norms. Evaluators of the second intervention did not have this experience before utilizing the RED checklist.

3. Time for Formal Feedback

Residents are busy and resident teams are often capped at full patient loads. They may be preoccupied with more pressing clinical duties that supersede teaching. We suspect residents could have rushed through grading and giving feedback to students compared to clerkship directors who had a dedicated period of time set aside to provide detailed student feedback.

4. The Social Aspects Influencing the Inpatient Teams Led to Score Inflation

We suspect a culture of politeness is present to avoid damaging a student’s self-esteem with constructive criticism [27]. Face-to-face evaluations of junior medical students resulted in grade inflation in one study [28]. Though these factors were likely present in both interventions, we hypothesize that there was a greater influence in the second intervention consisting of mostly resident evaluators due to the “social desirability bias” [29]. Since residents spend significantly more time with medical students throughout a clerkship and are near peers, they may be more inclined to give higher scores in an effort to build and maintain strong, interpersonal relationships with students.

Although clerkship director feedback was more effective than ward resident/attending feedback, our post-hoc analyses showed that the quality of medical student notes, as assessed by the RED checklist, significantly improved regardless of the source of feedback.

Our study has several strengths. Unlike past interventions, our study assessed real non-standardized inpatient progress notes written on wards. We had a large sample size consisting of 257 third-year medical students: 124 students (76%) in the 2017-2018 academic year and 133 students (85.8%) in the 2018-2019 academic year. A total of three different EHR systems (Epic, computerized patient record system (CPRS), and PowerChart) were used at the five different hospitals where students rotated, thus making these results generalizable to a variety of hospital systems.

This study was restricted to the Internal Medicine clerkship, and it is unclear if this intervention will improve student notes in other disciplines. Another limitation was the fact that interrater reliability was not measured. The greatest limitation of the study was a lack of a control group. We felt the feedback was critical to a student’s progress thus our study did not implement a control group that did not receive feedback. Future studies are needed to compare the note quality with feedback vs. no feedback. Further analysis comparing note quality scores on the same note when graded by a novice grader compared to an experienced grader is currently underway.

When attendings and residents give targeted feedback on notes, the quality improves. As obtaining feedback has been shown to be an integral part of medical education, the RED checklist can also help initiate the process of asking for and obtaining quality feedback from residents and faculty members. Many medical educators struggle to find time for providing structured quality feedback to trainees and lack tools to facilitate structured feedback. The RED checklist allows clinicians and residents to evaluate notes in seven to nine minutes without the need for any background information on patients. The use of various feedback methods and the nature of the tool allow the easy implementation of any clerkship or program. The RED checklist can also help provide students with clear expectations and guidelines on how to write a well-structured note. Furthermore, teaching hospitals nationwide should consider incorporating tools similar to the RED checklist to facilitate note feedback, as they begin to look for ways to evaluate the appropriateness of clinical notes written by students.

## Conclusions

In today's healthcare environment, clinical documentation plays a major role in patient care. Having accurate and clinically reasoned documentation is key for providing quality medical care. Our results showed significant improvement in student note quality as measured by the RED checklist after implementation of note feedback by either clerkship directors (intervention 1) or residents/attendings (intervention 2). As we continue to search for an effective and time-efficient method to teach note writing and improve student note quality, medical schools can consider implementing the RED checklist into their curriculum to facilitate note feedback and improve medical student documentation.

## Appendices

**Table 2:** Responsible Electronic Documentation (RED) Checklist Source: [18]

Subjective			
The note contains:	No (0)	Yes (1)	N/A
1. Current patient concerns or symptoms.			
The source may be the patient or family/caregiver.			
No = Does not include a symptom or state that the patient is without concerns. Yes = Indicates a symptom or that patient has no concerns.			
Total			
Objective			
The physical examination contains the following:	No (0)	Yes (1)	N/A
2. Succinct vitals.			
Succinct vitals are presented in a condensed way, not as a list of multiple sets from multiple time points.			
No = Vital signs from 3 or more time points are listed in full. Yes = Vital signs are succinct. N/A = There are no vital signs.			
3. Examination of all systems relevant to today’s positive symptoms.			
Look at the patient’s subjective symptoms. For each and all positive symptoms, at least one relevant organ system is examined. For vague symptoms such as pain, determine the site of pain and make sure that the organ system is included.			
No = All positive symptoms do not have at least one relevant organ system examined. Yes = For each positive symptom, at least one organ system is examined. N/A = There are no positive symptoms in the subjective.			
4. Examination different from the previous day’s exam.			
Disregard vital signs for this. Check that at least one change has been made to the exam. The change may be a statement acknowledging that the exam is unchanged. This statement needs to change day to day, (e.g. exam is unchanged from yesterday, March 7.)			
No = Examination is not different from the previous day’s exam. Yes = Examination is different from the previous day’s exam. N/A = There is no examination.			

Objective (cont.)					
The data portion of the note contains:	No (0)		Yes (1)		N/A
5. Labs only if they are new.					
Lab panels should be included only if they are new (i.e., the first day they are included). Trends should only be included if they are updated and every lab is relevant to patient care.					
No = Old labs without updates from prior notes are included, or trends are irrelevant. Yes = Only new labs and new and relevant trends are included. N/A = There are no labs.					
6. Reports of studies only if it is the first day they are included.					
Studies include ECGs, radiologic imaging, functional testing, procedures, and pathology reports.					
No = Old studies are included. Yes = Only new studies are included. Preliminary reports followed by final or modified reports of the same study on consecutive days are acceptable. N/A = There are no studies.					
Mark on the scale as defined in key:	0		1	2	N/A
7. A summary or impression of study reports.					
Studies include ECGs, radiologic imaging, functional testing, procedures, and pathology reports. The full report should not be included. Include new and old studies in your assessment.					
0 = For ANY study, the author has copied and pasted the report in excess of a summary or impression. 1 = All documented studies include only a summary or impression, but less than ½ of documented studies are in the author’s own words. 2 = ½ or more of studies are summarized in the author’s own words. N/A = There are no studies.					
Total					
Assessment and Plan					
The assessment and plan meet these criteria:	No (0)		Yes (1)		N/A
8. A summary statement is included.					
A summary statement is a snapshot overview distinct from the individual problem list.					
No = No or inadequate summary statement is included. Yes = Summary statement is included.					
9. The summary statement is different from the previous day’s statement.					
Any change is adequate. Even correcting a typo from the prior day reflects that the summary statement has been reviewed.					
No = The summary statement is identical to the previous day’s. Yes = The summary statement is different. N/A = There is no summary statement.					
Assessment and Plan (cont.)					
The assessment and plan meet these criteria:	No (0)		Yes (1)		N/A
10. Positive symptom(s) from the subjective section are included.					
Review the subjective section and identify all positive symptoms mentioned by the patient. Consider any medical symptom to be significant. Symptoms need not be distinct problems, but must be mentioned somewhere in the assessment and plan (A/P) or relate clearly to a problem that is in the A/P.					
No = Not all positive symptoms have a comment in the A/P. Yes = All positive symptoms have a comment in the A/P. N/A = There are no positive symptoms in the subjective section.					
11. A problem-based assessment is included					
The assessment is organized by problem, not listed by organ system (i.e., not cardiovascular, pulmonary, infectious disease, genitourinary, etc).					
No = A problem-based assessment is not included. Yes = A problem-based assessment is included.					
Mark on the scale as defined in key:	0	1		2	N/A
12. The status of each problem is described.					
Look at ALL items in the A/P. All problems should be described as improving, worsening, persistent, stable, resolved, new, or inactive.					
Include all problems except structural, hospital-specific paragraphs such as code status, dispo, contact info, prophylaxis. The terms acute or chronic do not fulfill the criteria for this item. Status of lab values (e.g., creatinine improving) do not fulfill criteria for this item unless the problem is the lab abnormality itself (e.g., hypokalemia worsening).					
0 = No problems are described as improving, worsening, persistent, stable, etc. 1 = Less than ½ of all problems are described as improving, worsening, persistent, stable, etc. 2 = ½ or more of all problems are identified as improving, worsening, persistent, stable, etc.					
13. Lab abnormalities are interpreted.					
Review NEW labs available in the note, and identify abnormalities of labs included in this list. Each individual lab (with the exception of fluid studies) should be commented upon either within another problem or as its own problem in the A/P. Each serum and culture result should be counted as an individual lab, whereas fluid studies should be counted in aggregate (e.g., CSF studies count as one lab).					
Abnormalities of labs not included in this list may exist and be commented upon in the A/P but are not part of the criteria for this item.					
CBC: Hemoglobin, white blood cell, platelet Chem 7: Sodium, potassium, bicarb, creatinine Misc chem: magnesium, phosphorous LFTs: AST/ALT, total bilirubin Coags: INR Cultures: any and all NEW results Fluid analyses: CSF, pleural, peritoneal.					
Assessment and Plan (cont.)					
Mark on the scale as defined in key:	0	1		2	N/A
13. Lab abnormalities are interpreted. (cont.)					
0 = None of the new abnormal labs from this list are mentioned in the A/P. 1 = Fewer than ½ of new abnormal labs from this list are mentioned in the A/P. 2 = ½ or more of new abnormal labs from this list are mentioned in the A/P. N/A = There are no new abnormal labs from this list.					
14. Interpretation of studies is included.					
Review NEW study results included in the note. Studies include ECGs, radiologic imaging, functional testing, procedures, and pathology reports. All studies with NEW results (including newly final or modified results) should be commented upon in the A/P, usually within a problem, even if the results are normal.					
0 = None of the new study results are mentioned in the A/P. 1 = Fewer than ½ of new study results are mentioned in the A/P. 2 = ½ or more of new study results are mentioned in the A/P. N/A = There are no new study results.					
15. Problems are written as diagnoses or accompanied by differentials.					
Look only at the first 3 items. The header for each paragraph should either be a disease or a symptom/abnormality with a differential (at least one possible diagnosis) easily identified under it. A diagnosed lab abnormality (e.g., hyponatremia) should include a cause or differential. Exclude structural, hospital-specific paragraphs such as code status, dispo, contact info, prophylaxis.					
0 = None of the first 3 items in the A/P is labeled as a disease (or symptom/abnormality with a differential). 1 = At least one of the first 3 items of the A/P is labeled as diseases (or symptoms/abnormalities with a differential). 2 = All of the first 3 items in the A/P are labeled as diseases (or symptoms/abnormalities with a differential).					
16. Active problems are accompanied by clinical reasoning.					
Look only at the first 3 items. Clinical reasoning is defined as any stated reason for either a plan or a diagnosis or assessment. Clinical reasoning can be brief. For example, “Pneumonia, improving. Fever curve downtrending.” Or “CHF, improving. Substantial diuresis.”					
0 = None of the first 3 items in the A/P are accompanied by clinical reasoning. 1 = At least one of the first 3 items in the A/P is accompanied by clinical reasoning. 2 = All of the first 3 items in the A/P are accompanied by clinical reasoning. N/A = There are no active problems.					

Assessment and Plan (cont.)				
Mark on the scale as defined in key:	0	1	2	N/A
17. Problems are associated with brief, clear plans.				
Look only at the first 3 items. Diagnostic and therapeutic plans should be brief and clear.				
0 = None of the first 3 items in the A/P have a diagnostic or therapeutic plan that is brief and clear. 1 = At least one of the first 3 items in the A/P has a diagnostic or therapeutic plan that is brief and clear. 2 = All of the first 3 items in the A/P have a diagnostic or therapeutic plan that is brief and clear. N/A = There is no plan.				
18. Assessment and plan are different from the previous day’s A/P.				
Look only at the first 3 items. A change may be a single word altered.				
0 = The first 3 items of the A/P are verbatim identical to the previous day’s A/P. 1 = At least one of the first 3 items in the A/P is changed from the previous day’s A/P. 2 = All of the first 3 items in the A/P are changed from the previous day’s A/P. N/A = There is no A/P.				
Total				

Summary – Faculty to complete	
A good progress note is TRUTHFUL, REASONED, UPDATED, AND SUCCINCT. Please comment on characteristics of this note that fulfill or lack these characteristics	
This note appears TRUTHFUL. Portions do not contradict one another or the prior note.	
This note is REASONED. It reflects rational clinical thought processes by the author.	
This note is UPDATED from the previous day’s note. It attempts to communicate the current state of the patient.	
This note is SUCCINCT. It is concise and easy to read.	

Subjective		Objective		Assessment & Plan		Total Note
	+		+		=	
